# Large contractors in Africa: conundrums with malaria chemoprophylaxis

**DOI:** 10.1186/s12936-016-1265-y

**Published:** 2016-04-12

**Authors:** Leo Braack

**Affiliations:** University of Pretoria Centre for Sustainable Malaria Control, Pretoria, South Africa

**Keywords:** Malaria, Acquired immunity, Prophylaxis, Intermittent preventive treatment

## Abstract

**Background:**

Despite high levels of naturally-acquired immunity (NAI) within local communities in malaria high transmission settings in Africa, such people often experience clinical disease during peak transmission months due to high parasite challenge. Major recruiters of unskilled labour in high-transmission malaria settings in Africa generally withhold chemoprophylactic medication from this large component of their labour force, which if administered during peak “malaria season” could reduce incidence of clinical malaria without unduly affecting NAI.

**Commentary:**

Naturally acquired immunity confers protection against severe clinical disease and death, but does not prevent mild clinical disease and, therefore, still results in worker absence and worker debilitation. Evidence exists that NAI persists despite periodic parasite clearance and therefore provides opportunity for drug prophylaxis during peak transmission months, which contributes to broader malaria elimination objectives, community well-being, and reduced absence from work. Such chemoprophylaxis could be by way of standard daily or weekly supervised administration of prophylactics during peak transmission months, or occasional intermittent preventive treatment (IPT), all aimed at reducing parasite burden and clinical disease. However, challenges exist regarding compliance with drug regimens over extended periods and high parasite resistance to recommended IPT drugs over much of Africa. Despite withholding chemoprophylactics, most large companies nevertheless pursue social responsibility programmes for malaria reduction by way of vigorous indoor residual spraying and bed net provision.

**Conclusions:**

The lack of clear understanding regarding functioning of NAI and its role in malaria elimination campaigns, concerns about drug resistance and appropriate drug choice, lack of studies in the use of IPT in people other than pregnant women and small children, plus lack of guidance regarding drug options for IPT in the face of widespread resistance to sulfadoxine–pyrimethamine, means that large contractors in malaria endemic settings will likely continue to withhold malaria prophylactic drugs from locally-recruited workers, with adverse consequences on workforce well-being. Nevertheless, if the point of chemoprophylaxis is to reduce clinical malaria by way of reducing parasite challenge without significantly impacting NAI, then a comparable result can be achieved by implementation of effective vector reduction programmes which minimize parasite transmission but maintain NAI.

## Background

Mining and other large, labour-intensive companies operating in remote areas of Africa often employ hundreds of local rural residents for unskilled labour. Medical staff at such facilities routinely make available or recommend malarial prophylactics to expatriate staff, but withhold it from local employees, which form the bulk of their establishment. This is not done for cost-saving reasons, but more usually as a precautionary measure out of concern for disrupting naturally-acquired immunity (NAI) among local people, particularly against the lethal and most common parasite *Plasmodium falciparum*; doubts about compliance with drug-taking regimens among workers is also a major factor in withholding such malaria prophylactic drugs. NAI does not prevent clinical malaria, but reduces the frequency and severity of such events [1]. However, the consequence of withholding malarial prophylactics is that the overwhelming majority of malaria cases annually recorded by such mining or similar operations occur within the segment of locally employed people. This in turn gives rise to concern among managers not just over lost production time, but also because they are confronted with questions why a relatively high percentage of staff contract malaria, which is a preventable disease.

Naturally acquired immunity is a complex phenomenon which is still not fully understood [1–3]. Nevertheless, as a general pattern, it is well-established that within populations living in holo- or hyperendemic areas of stable malaria transmission where parasite challenge occurs regularly, NAI develops to provide varying levels of protection from severe malarial disease and death. This is dependent on age and frequency of infection. Infants steadily lose maternally-acquired resistance to clinical disease by about 6 months of age, and from then are susceptible to severe malaria and high risk of death until acquiring self-induced resistance somewhere between age 2 and 5. Small children in areas of high parasite transmission develop resistance sooner than in areas of less intense transmission. By puberty, children experiencing heavy and largely uninterrupted exposure to infective mosquito bites rarely develop severe disease, although mild clinical episodes may still be common [1]. This state of resistance to severe disease and death by malaria is then more or less maintained throughout adulthood although pregnant women show susceptibility to severe clinical malaria and death—hence intermittent preventive treatment (IPT) routinely provided to pregnant women visiting health facilities [4]. NAI generally is also species-specific and even strain-specific, another reason why regular exposure to parasites is required to develop and maintain broad-spectrum immunity [1, 5]. It is important to note that NAI does not mean complete absence of *Plasmodium* parasites in the blood, but rather a state of sub-clinical asymptomatic disease with low persistence of parasites in the blood, in effect producing a constant immunological challenge and thereby maintaining NAI.

## Commentary

Telephonic interviews were conducted with medical representatives of six large international mining and construction operations in high transmission malaria settings in eight African countries (Table 1). Without exception, all indicated that they offered malaria prophylactics to non-immune (generally expatriate) staff, but as a rule such prophylactics are not offered to locally-recruited employees. Aside from general concerns about interfering with naturally acquired immunity, the single most frequent reason given for withholding prophylactics is the high level of poor compliance among workers in taking such drugs according to prescribed schedule (no attempt was made to enquire on what basis such conclusions were arrived at, whether by direct historic experience of the particular company or extrapolating from the experience of other companies, or mere assumption). Nevertheless, the primary motivating drive for withholding prophylactic drugs is a perception that the incidence of clinical malaria among workers would increase, and that withholding prophylactic medication from locally-recruited workers is therefore medically justified. The consequence of course, is that such a policy promotes the widespread presence of asymptomatic carriers of malaria parasites within the local community and encourages the formation of infective gametocyte stages within many such asymptomatic carriers, which infect mosquitoes and perpetuates the malaria cycle. At broader community level, therefore, NAI has the overall effect of *sustaining* malaria, despite the development of some transmission-blocking immunity reducing the efficiency of sexual stage gametocyte transmission to mosquitoes [6].

**Table 1:** Geographic distribution of companies interviewed and key question responses

	Country	High local malaria transmission, yes or no	Do you offer or recommend malaria prophylactics to expatriate staff?	Do you offer or recommend malaria prophylactics to locally-employed workers?	Do you engage in significant malaria control activities in surrounding communities?
Company A	Chad	Yes	Yes	No	Yes
Company B	Democratic Republic of the Congo	Yes	Yes	No	Yes
Company A	Ghana	Yes	Yes	No	Yes
Company A	Guinea	Yes	Yes	No	Yes
Company C	Mali	Yes	Yes	No	Yes
Company D	Mozambique	Yes	Yes	No	No
Company E	Tanzania	Yes	Yes	No	Yes
Company F	Zambia	Yes	Yes	No	Yes

The concerns of mining and similar major operations in malarious areas of Africa regarding the use of malaria prophylactics, and the unintended encouragement of community parasitaemia as a consequence, does not occur in a vacuum. Broader national and international interests are aimed at eradicating malaria and much success has been achieved in reducing malaria case numbers over the preceding decade [7]. The three predominant tools used in this globally coordinated campaign are provision of insecticide-treated bed nets (ITN’s), indoor residual spraying (IRS) of insecticides, and detection and treatment of infected persons using artemisinin-based combination therapy (ACT). The consequence of such national and international malaria control campaigns in theory works directly against the interests of maintaining NAI, firstly because ITN’s and IRS dramatically reduce the number of malaria-transmitting mosquitoes and therefore reduce exposure to parasites, and secondly because ACT clears malaria parasites in the blood and, therefore, also reduces exposure to parasites. In large areas of Africa where stable malaria transmission is the norm, regular exposure to malaria parasites is required to maintain NAI for optimum population well-being due to the effect of NAI in greatly reducing malaria mortality and morbidity [1].

The contradictions and complexities now become apparent. If people spending their lives in areas of high malaria transmission have NAI which provides them safety from severe clinical malaria and death, should the world then stop what might be misguided humanitarian efforts in eliminating malaria? Of course not. The most obvious reason is the very high fatality rate among children below 5 years of age as a direct consequence of malaria—despite the ameliorating effects of NAI—and also the debilitating impact of even mild malaria in subsequent years on child schooling plus the disproportionate impact on women by way of negatively affecting capacity for family care, income-generating activities and subsistence food production.

The apparent contradiction of countries engaging in malaria control and thereby potentially reducing NAI is probably not as counterproductive as may seem. Low levels of circulating parasites appear to be sufficient to maintain NAI and even periodic short-term clearance of infections is not necessarily bad [1, 8]; such lower levels of parasite challenge may benefit a person by reducing the number of clinical malaria episodes while still maintaining NAI [1]. Also, the estimated half-life of NAI leading to reduced susceptibility to clinical malaria is suggested to be in the order of about 5 years, while anti-parasite immunity that lowers parasitaemia has a half-life of approximately 20 years or even more [9]. This would explain why miners recruited in high-transmission southern African states can work at malaria-free South African mines for most of the year and return home for annual vacation without apparent increase in susceptibility to severe malaria or death back home. It also explains why IPT for pregnant women is perfectly acceptable, despite periodic complete clearance of parasites from the blood.

Further complicating the matter is the issue of ethics. However well-intentioned, a unilateral decision by any large international company to selectively prescribe healthcare within its workforce—especially when the decision is largely based on imperfect evidence and leads to one segment of workers being consciously allowed to contract disease—can expose that company to accusations of unfair discrimination. Clearly the issue is complex, but society demands resolution of even complicated situations with solutions that are equitable and not open to hints of benevolent patronage. That is the purpose of this paper, to explore ways in which such companies can fulfil their social responsibility in a fair and even-handed way.

One avenue for defensible compromise that addresses company concerns yet addresses societal expectations is to offer informed choice. Locally-employed workers could be given appropriate presentations by trusted local health workers to fully inform them of the options and then offered a choice. Those that opt for prophylaxis during peak transmission months could then be provided on-site supervised consumption of malaria prophylactic drugs to improve compliance. However, this means a daily or weekly regimen over a period of two or 3 months and again raises the same concerns within medical practitioners about the practicality of adequate compliance among workers who work shifts, take vacation or have days off, and sometimes even hide medication to hand to family members; poor compliance is a serious challenge that fosters development of drug resistance, a major global concern in the face of limited anti-malarial drug choices [10, 11].

If the primary objective of malaria chemoprophylaxis in local workers is to reduce the incidence of clinical episodes by reducing parasite challenge in peak transmission season and yet not unduly affecting NAI, then a more practical option is to implement IPT. This means administering a full therapeutic parasite clearance dose once or twice during the high transmission season to fully-informed, signed-consent local workers. The practice of malarial IPT is well-established and widely-applied among pregnant women and children [12–18] without rebound effect [14, 19] or negative impact on NAI [19]. The use of IPT will reduce parasite challenge within locally-recruited employees, diminish the likelihood of breakthrough into clinical disease and, therefore, reduce the number of malaria cases, without undue impact on NAI status. This will reduce loss of production time in companies and improve general well-being of local employees.

However, implementation of either of the two prophylactic options presented above—daily or weekly anti-malarials during peak transmission months or occasional IPT—will present the challenge “So which drug should we use?”. The historical preferred choice of chloroquine and pyrimethamine is no longer available due to near universal parasite resistance to these drugs, and even the current choice of sulfadoxine–pyrimethamine for IPT is rapidly approaching the end of its practical usefulness because of widespread resistance [18]. One possibility for IPT would be the combination of artemether–lumefantrine, but the efficacy of this drug combination in IPT remains untested. Yet again, a clear and unambiguous policy option for large contractors appears elusive.

Parasite reduction and lowered incidence of clinical malaria in local inhabitants of malaria high-transmission areas can nevertheless be achieved in other ways, without necessarily adversely impacting on NAI (Table 2). Many companies already engage in IRS and provision of bednets in local communities from which workers are recruited. This achieves a similar objective of reduced parasite transmission, but has wider impact and benefit at community level. Until greater clarity emerges regarding appropriate choices of malaria prophylactic drugs for IPT in high transmission settings, probably the best intervention for worker well-being and company benefit would be for large contractors to engage in malaria control activities that bring immediate and sustainable reduction in transmission intensity, such as IRS and provision of LLIN’s, which also complement and strengthen national government objectives of malaria reduction at community level.

**Table 2:** Descriptions of different immunological responses often discussed in malaria epidemiology (after Schofield and Mueller)

Clinical immunity	Acquired immunity that protects against clinical illness despite presence of malaria parasites in blood
Clinical tolerance	Lowered responsiveness to malaria parasite toxins
Anti-toxic immunity	Acquired immunity that neutralizes parasite toxins
Anti-parasite immunity	Acquired immunity that kills or inhibits growth or replication of malaria parasites

Use of such terminology in the scientific literature often overlaps, is sometimes used interchangeably, and can vary subtly in its various applications

## Conclusions

Taking all the above factors into consideration, it appears that medical practitioners at mining and similar companies in high-transmission African countries have considerable justification for not advocating malaria prophylactic drugs to locally-recruited staff as a general rule, although exceptions will exist such as immune-compromised persons with HIV/AIDS. Some proof that these mining and other large companies are not withholding malaria prophylactic medication as a mere cost-saving exercise is the near-universal practice of such companies to engage in other extensive and costly malaria control practices, in particular provision of insecticide-treated bednets and indoor spraying of residential quarters with mosquito-combatting insecticides. Such vector control programmes that result in reduced parasite transmission fundamentally achieve the same desired objective for locally recruited workers, that being to reduce parasite challenge during peak transmission season which in turn leads to reduced incidence of clinical malaria, but allows persistence of NAI. Nevertheless, the use of IPT as a more directly targeted method for periodic controlled parasite reduction in local communities deserves serious consideration for improved community well-being, provided appropriate anti-malarial drugs are available.

## Abbreviations

IPT: intermittent preventive treatment
IRS: indoor residual spraying of insecticidal products against walls of houses and dwellings
ITNs: insecticide-treated bed nets
NAI: naturally-acquired immunity

## References

[1] Doolan DL, Dobaño C, Baird JK (2009). Acquired immunity to malaria. Clin Microbiol Rev.

[2] Schofield L, Mueller I (2006). Clinical immunity to malaria. Curr Mol Med.

[3] Yazdani SS, Mukherjee VS, Chauhan VS, Chitnis CE (2006). Immune responses to asexual blood-stages of malaria parasites. Curr Mol Med.

[4] Menendez C (2006). Malaria during pregnancy. Curr Mol Med.

[5] Kinyanjui SM. The immunology of malaria. In: Okwa O, editor. Malaria parasites. InTech. 2012. ISBN:978-953-51-0326-4. http://www.intechopen.com/books/malaria-parasites/immunity-to-malaria-.

[6] Keegan LT, Dushoff J. Population-level effects of clinical immunity to malaria. BMC Infect Dis. 2013;13:428. http://www.biomedcentral.com/1471-2334/13/428. 10.1186/1471-2334-13-428 PMC3848694 24024630

[7] WHO. World malaria report 2014. Geneva: World Health Organization; 2015. www.who.int/malaria/publications/world_malaria_report_2014/en/.

[8] Taylor RR, Allen SJ, Greenwood BM, Riley EM (1998). IgG3 antibodies to *Plasmodium falciparum* merozoite surface protein 2 (MSP2): increasing prevalence with age and association with clinical immunity to malaria. Am J Trop Med Hyg.

[9] Filipe JA, Riley EM, Drakeley CJ, Sutherland CJ, Ghani AC (2007). Determination of the processes driving the acquisition of immunity to malaria using a mathematical transmission model. PLoS Comput Biol.

[10] Greenwood B (2004). The use of anti-malarial drugs to prevent malaria in the population of malaria-endemic areas. Am J Trop Med Hyg.

[11] WHO (2010). Global report on antimalarial efficacy and drug resistance: 2000–2010.

[12] Cissé B, Sokhna C, Boulanger D, Milet J, Bâ EH, Richardson K (2006). Seasonal intermittent preventive treatment with artesunate and sulphadoxine–pyrimethamine for prevention of malaria in Senegalese children: a randomised, placebo-controlled, double-blind trial. Lancet.

[13] Kayentao K, Kodio M, Newman RD, Maiga H, Doumtabe D, Ongoiba A (2005). Comparison of intermittent preventive treatment with chemoprophylaxis for the prevention of malaria during pregnancy in Mali. J Infect Dis.

[14] Macete E, Aide P, Aponte JJ, Sanz S, Mandomando I, Espasa M (2006). Intermittent preventive treatment for malaria control administered at the time of routine vaccinations in Mozambican infants: a randomized, placebo-controlled trial. J Infect Dis.

[15] Shulman CE, Dorman EK, Cutts F, Kawuondo K, Bulmer JN, Peshu N (1999). Intermittent sulphadoxine–pyrimethamine to prevent severe anaemia secondary to malaria in pregnancy: a randomised placebo-controlled trial. Lancet.

[16] Van Eijk AM, Ayisi JG, ter Kuile FO, Otieno JA, Misore AO, Odondi JO (2004). Effectiveness of intermittent preventive treatment with sulphadoxine–pyrimethamine for control of malaria in pregnancy in western Kenya: a hospital-based study. Trop Med Int Health.

[17] Wilson AL (2011). On behalf of the IPTc Task Force. A systematic review and meta-analysis of the efficacy and safety of intermittent preventive treatment of malaria in children (IPTc). PLoS One.

[18] White NJ (2005). Intermittent presumptive treatment for malaria. PLoS Med.

[19] Schellenberg D, Menendez C, Aponte JJ, Kahigwa E, Tanner M, Mshinda H, Alonso P (2005). Intermittent preventive treatment for Tanzanian infants: follow-up to age 2 years of a randomised, placebo-controlled trial. Lancet.

